# Quantifying the Onset and Progression of Plant Senescence by Color Image Analysis for High Throughput Applications

**DOI:** 10.1371/journal.pone.0157102

**Published:** 2016-06-27

**Authors:** Jinhai Cai, Mamoru Okamoto, Judith Atieno, Tim Sutton, Yongle Li, Stanley J. Miklavcic

**Affiliations:** 1 Phenomics and Bioinformatics Research Centre, University of South Australia, Mawson Lakes, SA 5095, Australia; 2 Australian Centre for Plant Functional Genomics, University of Adelaide, Hartley Grove, Urrbrae SA 5064, Australia; 3 South Australian Research and Development Institute, 2b Hartley Grove, Urrbrae SA 5064, Australia; Mediterranean Agronomic Institute at Chania, GREECE

## Abstract

Leaf senescence, an indicator of plant age and ill health, is an important phenotypic trait for the assessment of a plant’s response to stress. Manual inspection of senescence, however, is time consuming, inaccurate and subjective. In this paper we propose an objective evaluation of plant senescence by color image analysis for use in a high throughput plant phenotyping pipeline. As high throughput phenotyping platforms are designed to capture whole-of-plant features, camera lenses and camera settings are inappropriate for the capture of fine detail. Specifically, plant colors in images may not represent true plant colors, leading to errors in senescence estimation. Our algorithm features a color distortion correction and image restoration step prior to a senescence analysis. We apply our algorithm to two time series of images of wheat and chickpea plants to quantify the onset and progression of senescence. We compare our results with senescence scores resulting from manual inspection. We demonstrate that our procedure is able to process images in an automated way for an accurate estimation of plant senescence even from color distorted and blurred images obtained under high throughput conditions.

## Introduction

Even though image processing and computer vision methods have been applied in a range of plant biology contexts and over a span of years [1–6], the use of these techniques in a fully automated and high-throughput setting is still being established. This applies particularly to the topic addressed in this paper: the automated phenotypic analysis of leaf senescence, one of the trademark indicators of plant age and ill health.

Leaf senescence is the integral response of leaf cells to the regular ageing process but also to unfavorable environmental conditions [7]. Many physiological, biochemical, and molecular studies of leaf senescence [7–11] have shown that during senescence, leaf cells undergo highly coordinated changes in cell structure, metabolism and gene expression. The earliest and most significant change is the breakdown of chloroplasts; leaf senescence leads to the degradation of photosynthetic pigments such as chlorophyll, with the degradation manifested in observable leaf colour changes from the usual deep green to pale green, to yellow and finally to brown. Given our ability to observe these visual cues, it would be natural to consider the use of image processing techniques for a high-throughput plant leaf senescence analysis.

In this respect it would seem reasonable to consider employing the Normalized Difference Vegetation Index (NDVI), which indeed has been widely used for vegetative studies to estimate crop yield, pasture performance as well as plant senescence [12, 13]. However, this measure is sensitive to many factors including soil condition and water content [14]. This sensitivity limits the reliable and practical use of NDVI to the detection of vegetation coverage. With the introduction of hyperspectral imaging, a richer variety of quantitative measures (vegetation indices) is possible and indeed has already been introduced [15, 16] to quantify leaf traits (such as chlorophyll content, detection of leaf disease symptoms or indeed senescence determination). The simplex volume maximization concept [15] appears particularly promising for drought stress detection. A particularly recent technological development is HyperART [16], a hyperspectral imaging system which utilizes both reflectance and transmittance information to determine an absorption spectrum, used to estimate leaf chlorophyll content. One key innovation with HyperART lies in its ability to non-destructively scan an entire leaf still attached to a plant. This represents an advance on previous methodologies which were limited to point measurements (scan area of a few cm^2^) and being of lower resolution. Despite advances such as HyperART, the state of the art technology (compounded by the practical problem of cost effectiveness) is not yet geared for high throughput phenotyping applications on the whole plant scale (or whole canopy scale). With NDVI unsuitable for senescence analysis and with visual inspection [13], even by trained inspectors, being slow, weakly quantitative and prone to human subjectivity, and until high throughput hyperspectral phenotyping becomes viable, a need exists in the interim for the application of non-destructive and fully automated RGB image-based techniques for the objective estimation of plant senescence.

**Fig 1:**
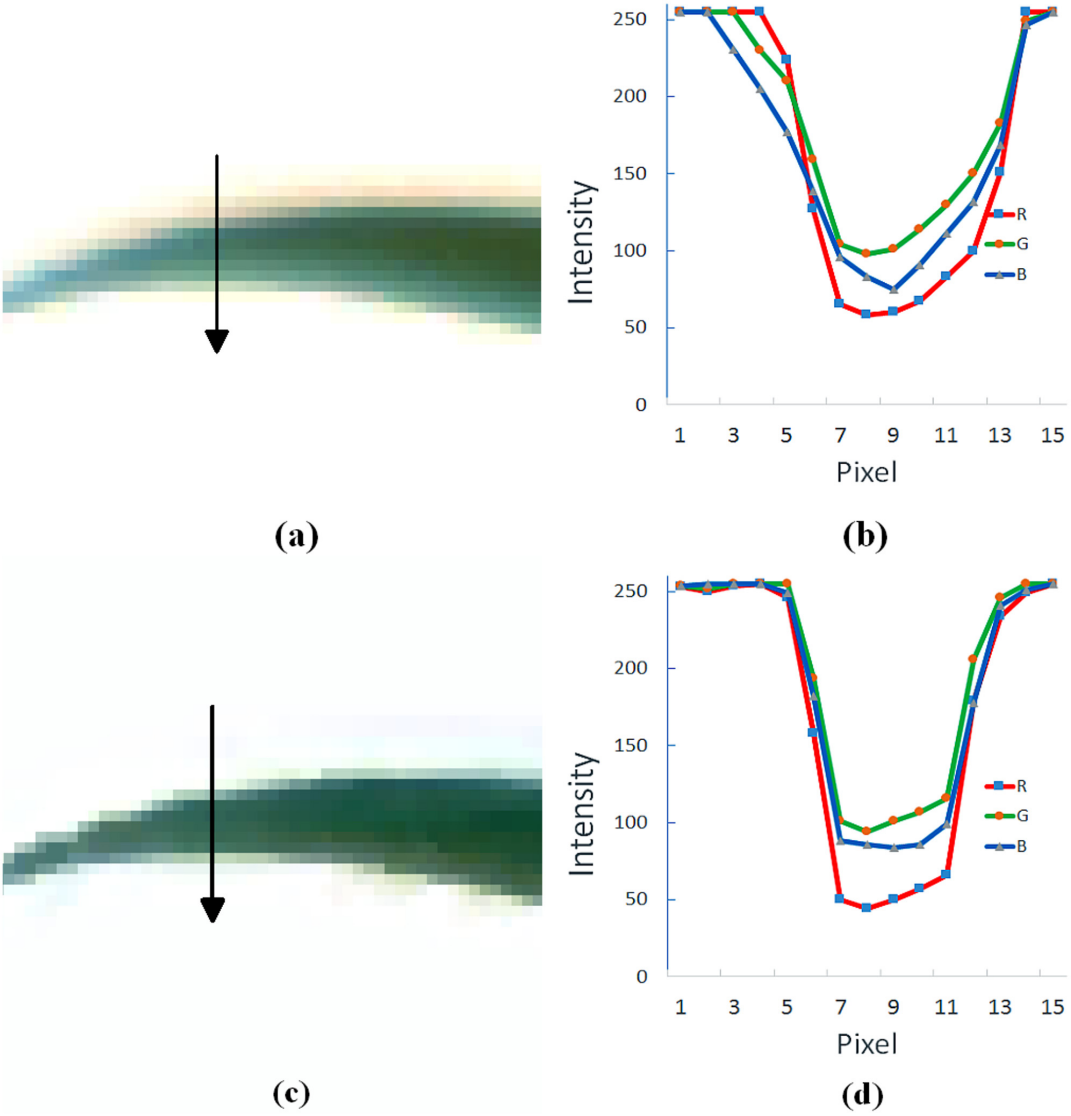
The color distortion effect and its correction using the method presented in this paper. (a) original image of a young and green leaf; (b) the corresponding color profile at the cross-section indicated by the arrow in (a); (c) image of the same leaf after color distortion correction; (d) corresponding color profile at the exact same cross-section.

Ideally, using high definition, high resolution RGB images at the leaf level, one could attribute leaf image color into a few categories of classification and use the ensuing full color distribution to estimate the senescence level of an entire plant. However, for the high-throughput practices we envisage, it is not feasible to take high definition images of all individual plant leaves. Instead, one resorts to taking a single image, or at most a few images from different perspectives, of a whole plant, which are then analyzed to determine the degree of senescence at the whole plant level. Under these pragmatic conditions, even if the global image resolution is high, the local resolution, at the leaf level, may not be. For example, Fig 1 shows an image of a young plant that in reality exhibits no actual senescence, but based on the image itself (a target for an automated procedure) would be assessed as already exhibiting senescence. Consequently, it can be problematic to apply a color classification scheme as such images may suffer from significant image blurring and therefore significant image color distortion, *at the level of an individual leaf*. It is an inescapable fact that the color of a pixel in a blurred image is affected by the colors of neighboring pixels [17]; the color of a pixel in a blurred image would then not represent the true color of an object feature at that specific location. For the analysis of plant senescence, this has unfavorable implications: the application of an image-based, color classification scheme could result in significant error. Consequently, a deblurring or restoration stage is required to reduce the extent of color distortion in such blurred images.

In this paper, we propose a new approach for color distortion correction in blurred images for the specific purpose of analyzing plant senescence. While this is important for the accurate quantification of senescence over the lifetime of the plant, it is absolutely essential for the purpose of pinpointing the time at which senescence in a plant first appears. Both aspects are important phenotypic traits. The approach we adopt, described in the next section, does not assume any of the currently adopted circumstances (color channel independence and point spread function (PSF)-based model de-blurring). However, we do make the reasonable and practical assumption that during the course of an experiment, or for a given image data set to be analyzed, the camera settings remain fixed. That is, we assume that the effects of blurring and color distortion remain consistent across all images acquired in a complete experiment. With this reasonable assumption of constant camera settings, we can take advantage of a priori knowledge of the conditions of the experiment to develop an algorithm for the correction of color distortion. In the results section we demonstrate and discuss the performance of our correction and analysis approach through applications to two time series of images for two different plant types, wheat and chickpea plants. We make concluding comments in the final section, where we point out that the proposal has the potential for a broader range of applications to quantify other phenotypic traits based on color discrimination.

## Materials and Methods

### Plant material and growth condition

**Wheat:** Australian spring wheat (*Triticum aestivum*) cultivars Gladius and Kukri were grown in pots in glasshouse conditions between January and June, and between August and December, 2013. Preselected similar sized seeds were sown in pots filled with 2.5kg of soil mix (coco-peat based potting media containing different amounts of nitrogen (N)). Nitrogen as urea was applied at sowing at 10mg (N1), 25mg (N2), 75mg (N3), 150mg (N4), and 450mg (N5) N/kg of soil. Plants were grown and watered in a glasshouse with average temperatures ranging between 22°C during the day and 15°C at night. At four weeks, the plants were transferred into a special growth room at the Australian Plant Phenomics Facility, University of Adelaide, Australia (The Plant Accelerator) for regular automated imaging using a LemnaTec imaging system (LemnaTec GbmH, Aachen, Germany). RGB images were automatically captured daily for 21 days.

**Chickpea pilot experiment**: Plant material consisted of two *Cicer arietinum* genotypes (Genesis 836 and Rupali). Experiments were again conducted in The Plant Accelerator. Temperature and humidity were controlled in the glasshouse and ranged from 24±2°C, 40% (day) and 16±2°C, and 90% (night), respectively. Three seeds were sown 2 cm deep in pots containing Goldilocks mix (50% clay loam, 25% University of California (UC) mixture, and 25% cocopeat). Rhizobium inoculum was added to each planting hole at sowing. Prior to salt application, plants were uniformly thinned to 1 plant per pot. At 25 days after sowing (DAS), each pot received either 0, 30, 40 or 60 mM NaCl. Each treatment was replicated 4 times in a Randomized Complete Block Design (RCBD). Pots were watered and maintained at field capacity to maintain the salt concentration and to avoid salt leaching.

### Imaging and manual senescence scoring

To allow for quantification of onset and to track the progression of plant senescence (chlorosis and necrosis), plants were imaged from 18 DAS up to 39 DAS. For each plant, RGB images were taken automatically from three different views (one top and two side views, the latter with a relative rotation of 90°).

To establish a correlation between visual scoring and digital image estimation of plant senescence, visual scores of the plants were taken based on a 1–10 scale [18] at 41 and 42 DAS.

**Scoring scale:**
1 = A green and healthy plant with no symptoms of illhealth (e.g., salinity stress);
2 = Bottom leaves beginning to yellow or become necrotic;
3 = Necrosis on a quarter of bottom leaves (25%) and yellowing on the rest of the bottom half of the plant;
4 = Necrosis on bottom half (50%) of plant;
5 = Necrosis on bottom half and yellowing appearing in the top half of the plant;
6 = Necrosis in the range 50%–75% of the plant;
7 = Necrosis on 75% of the plant;
8 = Necrosis on the whole plant with apical leaves still green/yellowing;
9 = Only stems and shoot tips remain green;
10 = Plant death.

### Modeling color distortion

Deblurring has been a subject of intensive study for decades [19]. Most deblurring algorithms developed thus far focus on estimating so-called shift-invariant point spread functions (PSFs) [20], under the assumption that blurring is caused either by the relative motion of the camera-object system, camera defocussing or by lens aberrations [21]. Recent research has also included the study of images blurred as a result of large depth differences (caused mainly by the limited focal depth of cameras). In this case, the focus is placed on estimating the PSFs [19, 21, 22] to produce better quality images, similar to those produced by an ideal pin-hole camera, except possibly for “ringing artifacts” [17, 20], a problem yet to be solved.

One assumption commonly adopted in previous works on deblurring is that the different spectral bands of visible light have the same properties, which implies that these spectral bands have identical PSFs. A second assumption is that of no interference between spectral band signals detected by different image sensors. Unfortunately, as Fig 1 indicates, these assumptions are not valid in the present context. Fig 1(a) is (actually) an image of a healthy green leaf of a young wheat plant. The image itself, however, clearly possesses significant yellowness around the leaf edges, this color distortion effect is quantified in Fig 1(b). The latter figure demonstrates that the three spectral bands are affected to different degrees and thus have different PSFs. Moreover, the curves appear asymmetric suggesting that they are not amenable to modelling by PSFs at all. In summary, existing deblurring algorithms are not applicable and indeed do not produce restored images of suitable quality. To improve the quality of restored images for an accurate estimation of plant senescence requires a less restrictive approach.

The task of deblurring an image is commonly called image deconvolution. If the blur kernel is not known, the problem is referred to as blind image deconvolution. Many methods have been proposed for deblurring from a single image [23, 24]. Existing blind deconvolution methods assume that the blur kernel has a simple parametric form, such as a Gaussian or a composition of low-frequency Fourier components [25].

In our procedure, we define the blurring degradation process by the expression

$$\begin{array}{c} X=GD+N, \end{array}$$ (1)

where **X** is the observed or degraded image, **D** is the degradation matrix or PSF, **G** is the image without degradation or blurring, and **N** denotes noise in the observed image. Note that we do not assume that any two spectral bands have the same PSF (**D**), nor do we assume that the PSF of a particular spectral band is independent of other spectral bands. In contrast to established methods where knowledge of the PSF is essential, in the formulation represented by Eq 1, the PSF is not invertible as it is inhomogeneous and multi-channelled. In the approach proposed here, we attempt to estimate directly the color distortion correction matrix (**C**) defined in the deblurring process, expressed mathematically as,

$$\begin{array}{cc} & G=(X-N)C,\\ \text{or} & \;\\ & E=G-XC. \end{array}$$ (2)

The matrix **C** denotes the matrix for the color distortion correction and **E** is the estimation error for the given level of noise. By minimising the mean square error, we can estimate the correction matrix using knowledge of both the undistorted and the observed images

$$\begin{array}{c} C={\left(X^{t}X\right)}^{-1}X^{t}G, \end{array}$$ (3)

where **X**^*t*^ denotes the transpose of the matrix **X** and **X**^−1^ denotes the inverse of **X**. In Eq (3), we assume that at least one *undistorted image* **G** is available for the estimation of **C**.

### Estimation of ground-truth color

In a pragmatic sense, the problem of estimating the color distortion correction matrix in Eq (1) reduces to the problem of obtaining at least one undistorted image for the subsequent application of Eq (3) to all distorted images assuming the same camera settings. The solution of the reduced problem could form part of an initialization step, being camera calibration; a standard color chart can be used to reconstruct an undistorted image given that the colors of the individual pixels of the color chart image are known. However, in the possibly more common event that camera calibration has not been attempted, with the notional consequence that an undistorted image cannot be reconstructed, it is still possible to estimate the undistorted image from the distorted image.

In the current application (which can be modified to suit other applications) we rely on the premise that plant leaves are green at the early stage of plant growth and development, particularly under advantageous conditions (*i.e.*, under no stress) to infer that any yellowness appearing in images of leaf edges is due solely to color distortion. As with conventional blind deblurring approaches, we exploit the information from the edges of two objects. We observe that the red channel signal in Fig 1(a) is sharpest at the edges of leaves. With this feature, the red channel of an image of a young plant can be used in an initial segmentation attempt in order to estimate the undistorted image of the young plant, for which leaf and stem color is unvarying across the whole plant. In this specific application we can estimate the real color at a leaf edge by the color of the interior section of that leaf image. We also manually select simple background regions of one image of the training set to estimate the true background color at boundaries between the plant and the background (and in this instance also between the blue plant support frame and the background). As there is color distortion in the original image, the initial segmentation attempt based on the red channel signal alone is not perfect. To improve the result, a manual correction is performed on this initial segmented image.

**Fig 2:**
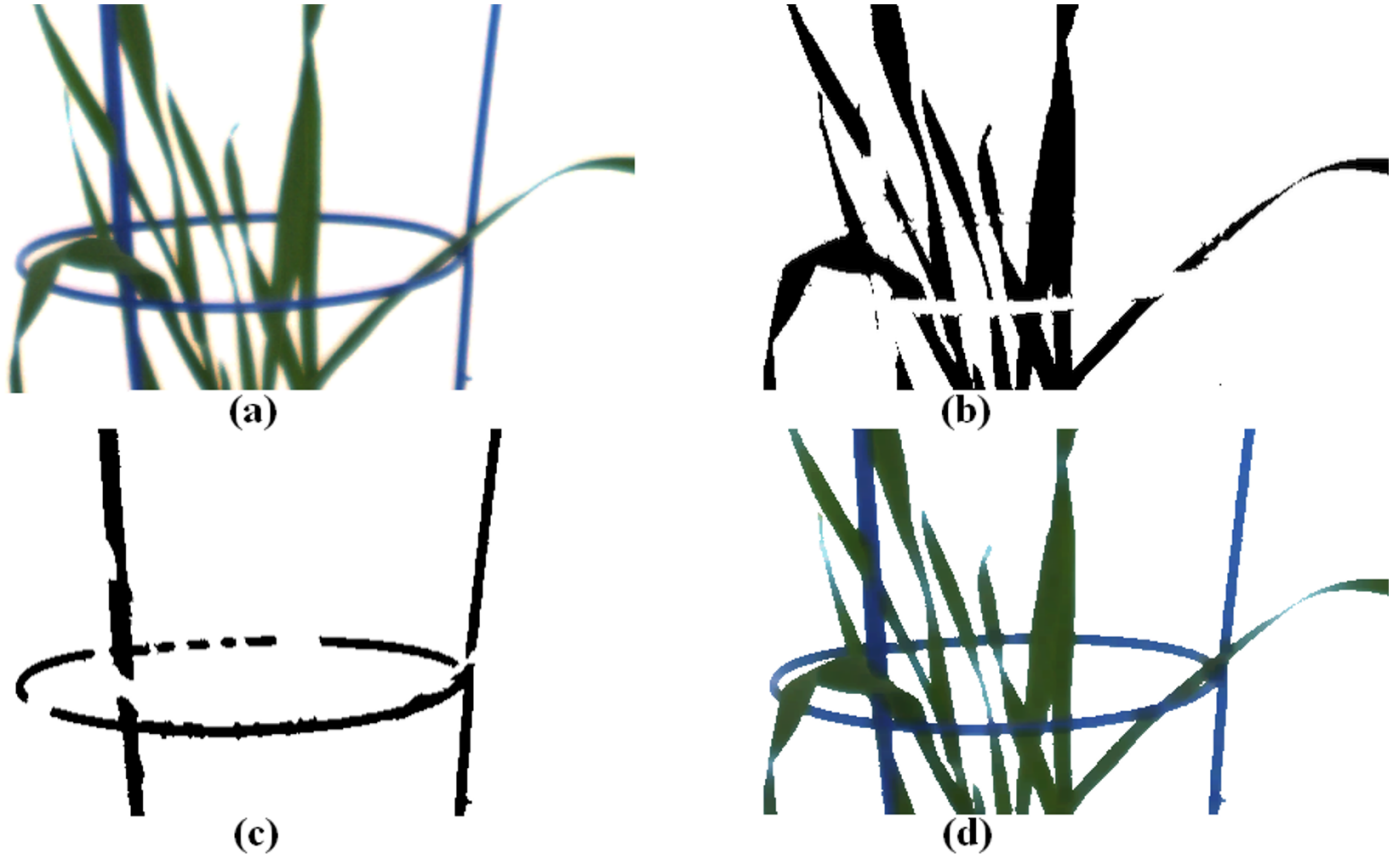
Example results at the end of each step in the process of ground-truth color estimation. In sequence: (a) the original image of a young plant; (b) the automatically segmented plant leaves with manual correction; (c) the automatically segmented frame with manual correction; and (d) the estimated image of the young plant after color distortion correction.

The results at the end of each step in the sequence of ground-truth color estimation are illustrated in Fig 2. A tell-tale sign that the initial attempt at color estimation is imperfect, is the bluish appearance of the leaf tips and of leaves of only a few pixels width.

### Color distortion correction

Now that an undistorted image has been obtained, we can use Eq (3) to estimate the correction matrix **C**. However, any two-dimensional image with multiple color channels cannot be directly represented by a single two-dimensional matrix. If, on the other hand, we assume that each channel is independent of others, Eq (3) can be directly used to deduce the correction matrix for each channel. Unfortunately, different color sensor cells corresponding to a given pixel are physically close to each other in the sensor panel. Therefore, it is possible that blurring effects are not channel independent. To treat the general case, we consider the correction matrix as a *M* × *N*_*c*_ matrix, where *M* = *L*^2^ × *N*_*c*_, *L* is the kernel size of the correction matrix, and *N*_*c*_ is the number of color channels, usually with *N*_*c*_ = 3. Furthermore, we arrange the estimated undistorted image into a *S*_*i*_ × *N*_*c*_ matrix, where *S*_*i*_ is the image size, *i.e.*, the total number of image pixels. We arrange the original, distorted image into a *S*_*i*_ × *M* matrix, which means that all pixels within the kernel are included for a given position. With this formulation, we make no explicit assumption about the correction matrix. The only disadvantage with this formulation is that the resulting size of matrix **X** is considerable making the calculation of **C** slow. Fortunately, the calculation of **C** is only required once for an entire experiment.

The final color distortion corrected matrix, **R** (the restored image), is obtained by a simple matrix multiplication: **R** = **XC**.

## Results and Discussion

### Analysis of the color distortion correction

**Fig 3:**
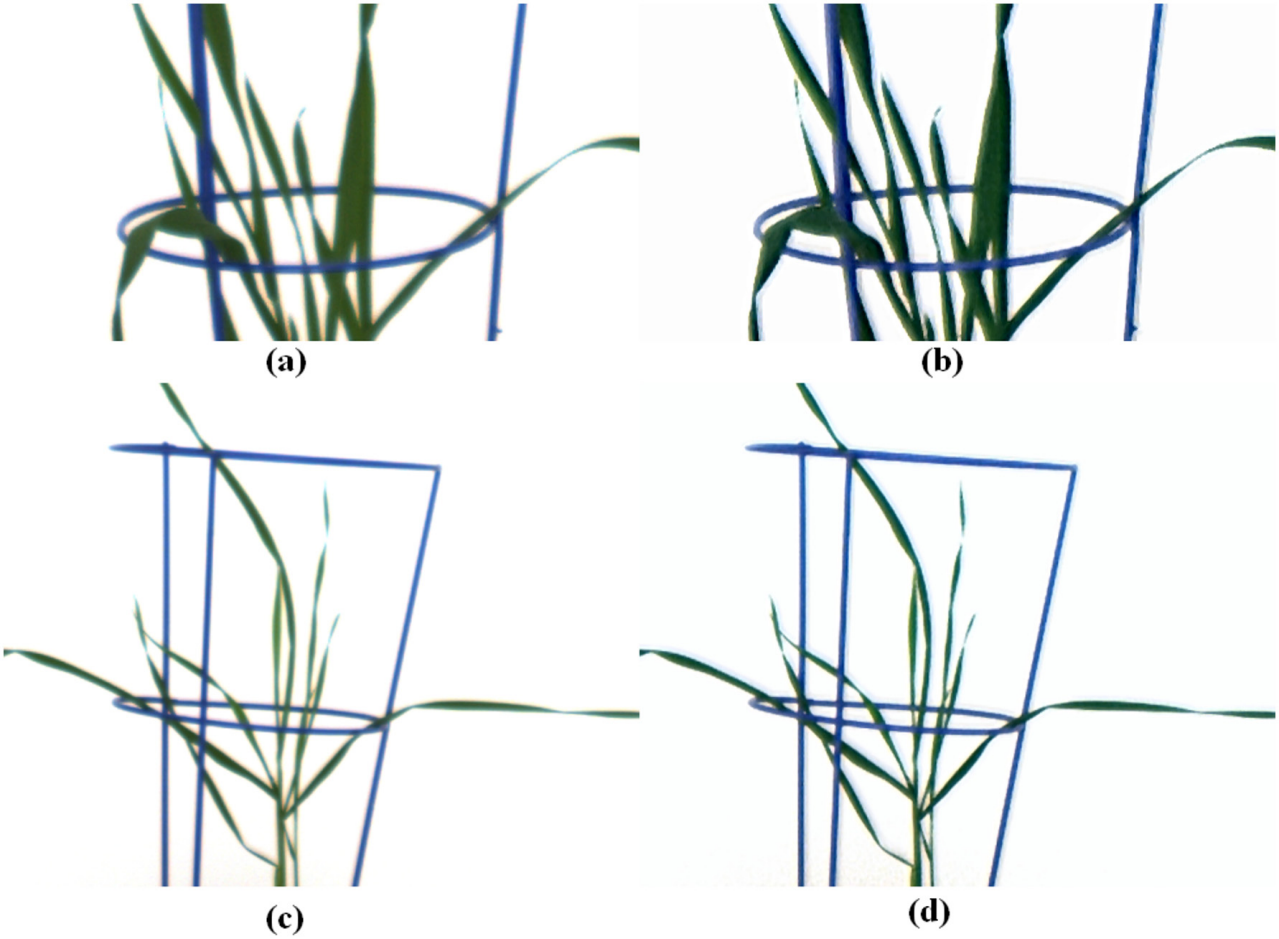
Result of a color distortion correction as applied to a young (non-senescent) plant. (a) the original image used for training;(b) the restored image after color distortion correction from (a); (c) the original image and (d) the restored image after color distortion correction from (c).

Given the absence of actual ground-truth information and given that a correction matrix is constructed from distorted data, it is prudent to first assess the performance of our approach based on an image of a young plant that we have used for training. Comparing Fig 3(a) with Fig 3(b) and Fig 3(c) with Fig 3(d), the restored images are sharper than the original images and the problem of yellowish tinge between green leaves has been significantly reduced. Confirmation of the effectiveness of the scheme can be derived from Fig 1(c) and 1(d), which highlight the improvement in color representation of the single leaf in Fig 1(a) and the effect on the color intensity profiles over the lateral cross-section indicated. The key feature of Fig 1(d) to note is the increased sharpness in the intensity changes across the boundaries of the leaf, now consistent across all three color channels. Admittedly, the process has introduced ringing artifacts [17, 20] in the restored images. As in the case of ringing resulting from deblurring algorithms, this problem has yet to be solved. Fortunately, the color possessed by the artifacts is bluish, which is thus distinct from the color of either green or senescent leaves. Therefore, such artifacts do not affect our senescence analysis.

**Fig 4:**
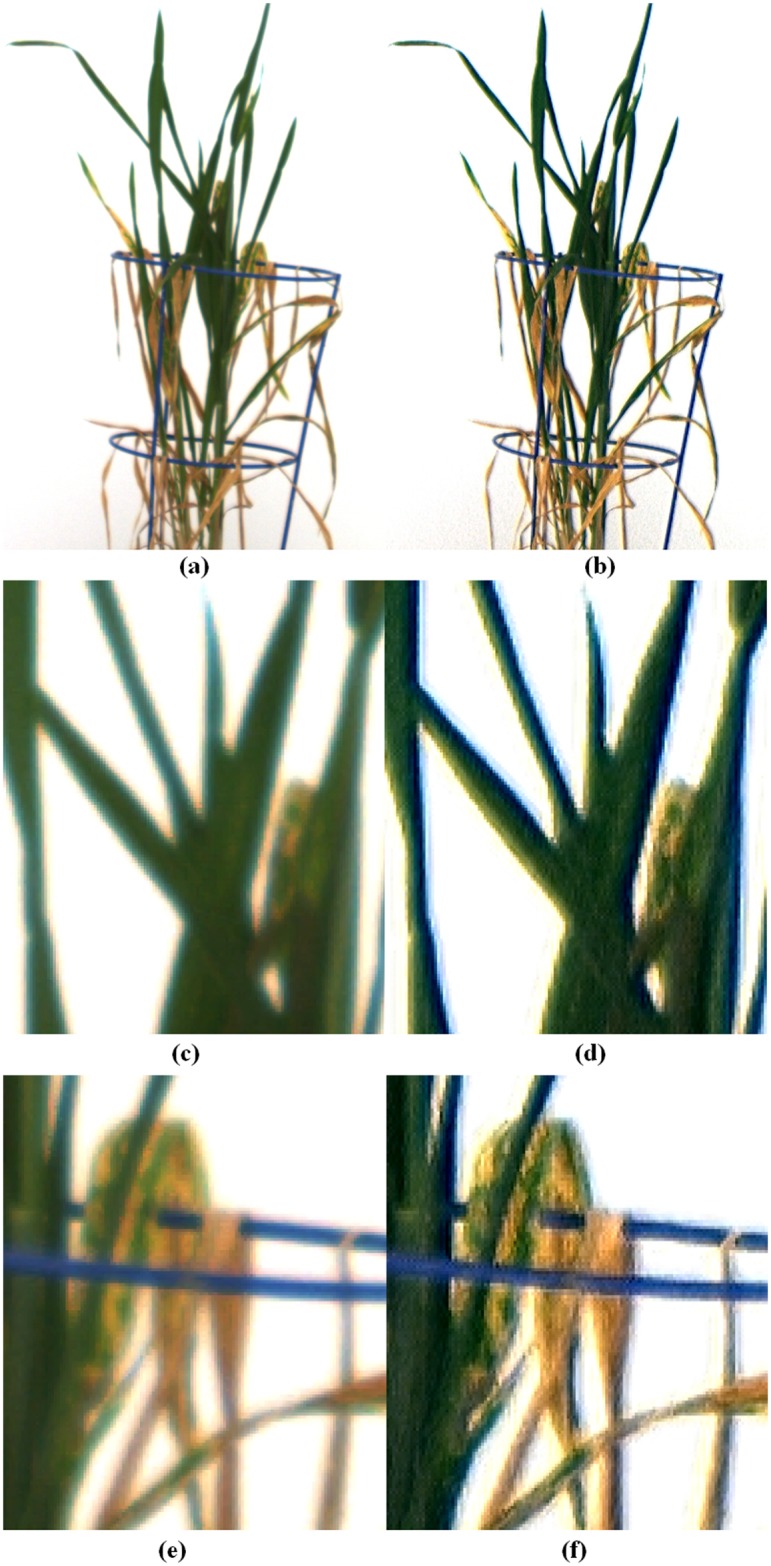
Results of application of the color correction process on plants exhibiting senescence. (a) shows the original image, while (b) shows the post processed, restored image. Panels (c) and (e) are enlarged regions of the original image in (a), while (d) and (f) are corresponding enlarged regions of the restored image (b).

With regard to images of plants with both green and yellow leaves, the restored image in Fig 4(b) has sharper edges than the original image (Fig 4(a)). Comparing Fig 4(c) with Fig 4(d), a significant amount of the blurred background area with characteristic yellow has been removed; such areas would lead to an overestimation of leaf senescence. Indeed it can be concluded from Fig 4(e) and 4(f) that our procedure does not affect the coloration of senescent leaves. In fact, the opposite appears to be the case, the color contrast between the green and the senescence regions of leaves is enhanced, which only benefits a color classification assessment. Although our procedure corrects for color distortion and enhances resolution and color contrast, the procedure is not perfect, as evidenced by the traces of yellow tinge found at leaf tips and corners of leaf image overlap.

### Senescence analysis

**Fig 5:**
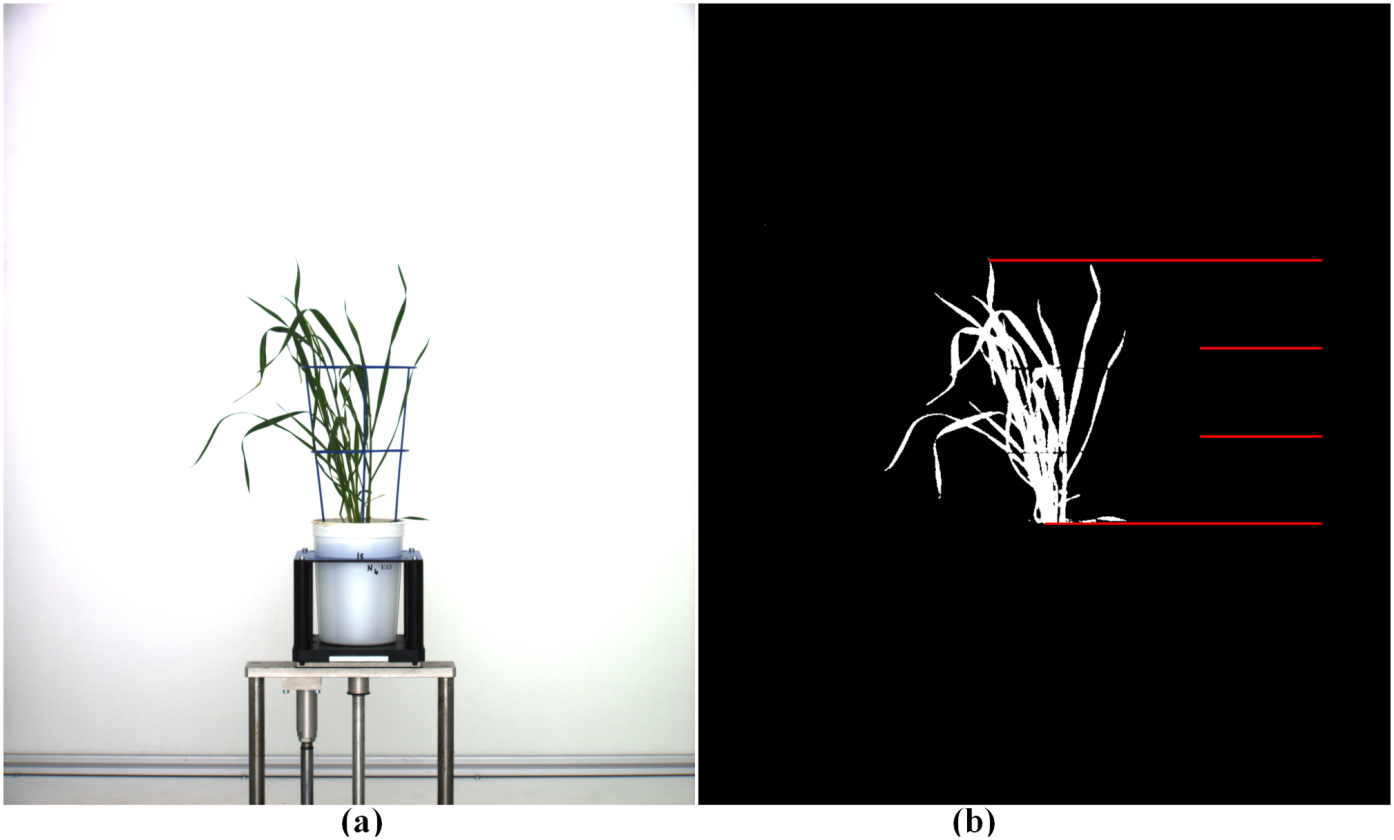
Demonstration of the image segmentation process and zonal partitioning of plant foreground. (a) is the original image, while (b) shows just the segmented plant image with overlayed horizontal lines partitioning the image foreground into three zones.

To analyze leaf senescence in plants, we first separate plant objects from background and then divide the segmented plant image into three vertical regions (zones) equidistant in height, as illustrated in Fig 5 (see also S1 Appendix). In each zone, the colors are classified into four categories: dark green, light green, light yellow and brown. Any part of a leaf with yellow or brown color is classified as undergoing or having succumbed to senescence. Note that any leaves that have dropped below the bottom line are assigned to the bottom zone.

#### Wheat experiment

**Fig 6:**
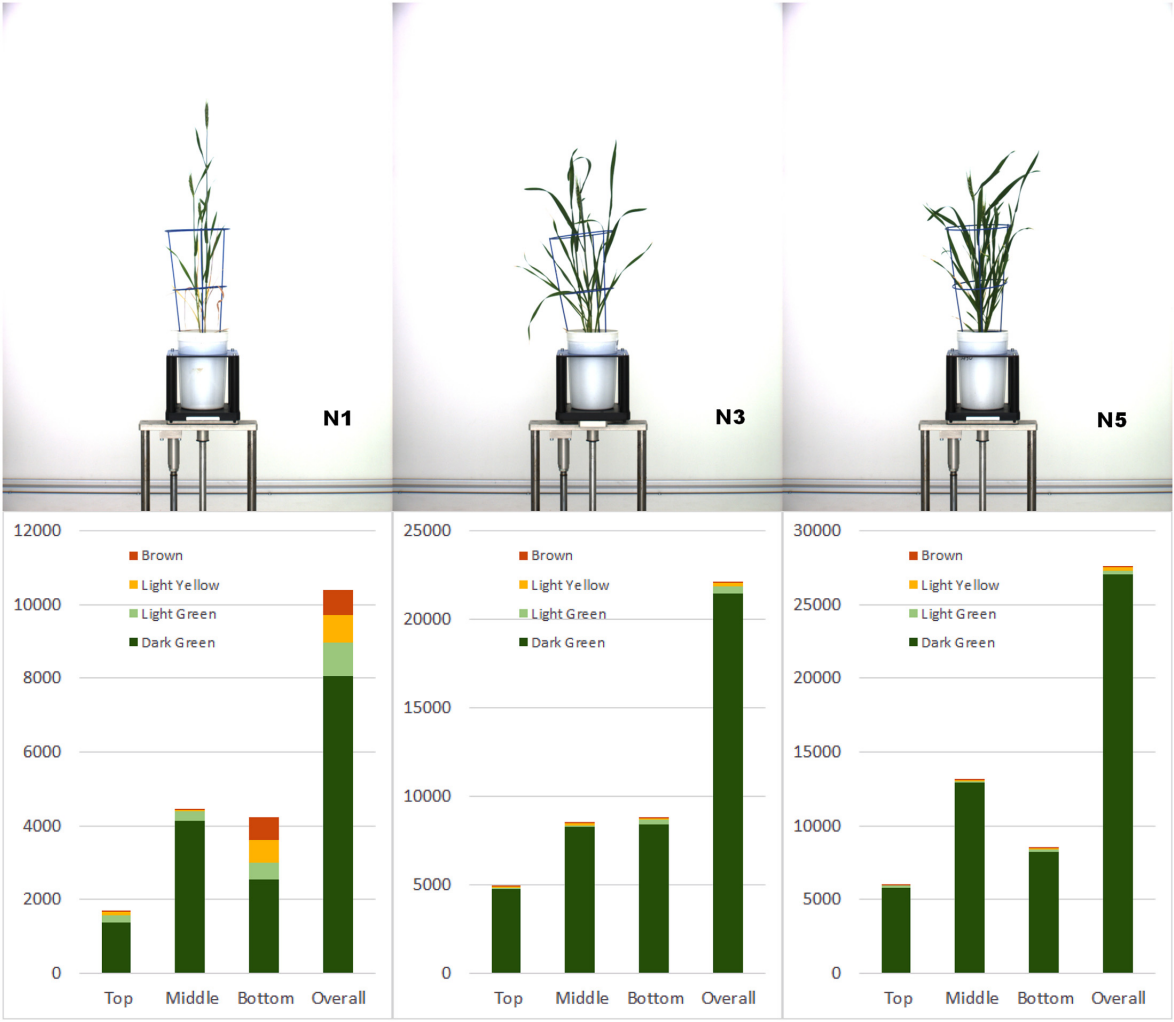
Zonal assessment of green versus senescence leaf areas in pixels. Top figures show original images of three Gladius plants each grown under a different level of nitrogen (N1, N3 and N5). The bottom figures show the results of color classification in pixel area as a function of zone, according to our four-category color scheme: dark green, light green, yellow and brown.

Our procedure (as well as a color classification analysis) was applied to images of wheat plants to assess the affect of nitrogen availability on the development of leaf senescence. A full biological analysis of this experiment will appear elsewhere. For the purposes of this paper, we show in Fig 6 examples of the color analysis on images of three plants, each exposed to a different level of nitrogen (low, medium and high, respectively). The top row of figures shows restored images of the plants as they appeared on the same day. The bottom row shows the corresponding four-category, color classification assessment as a function of zone as well as measured overall.

**Fig 7:**
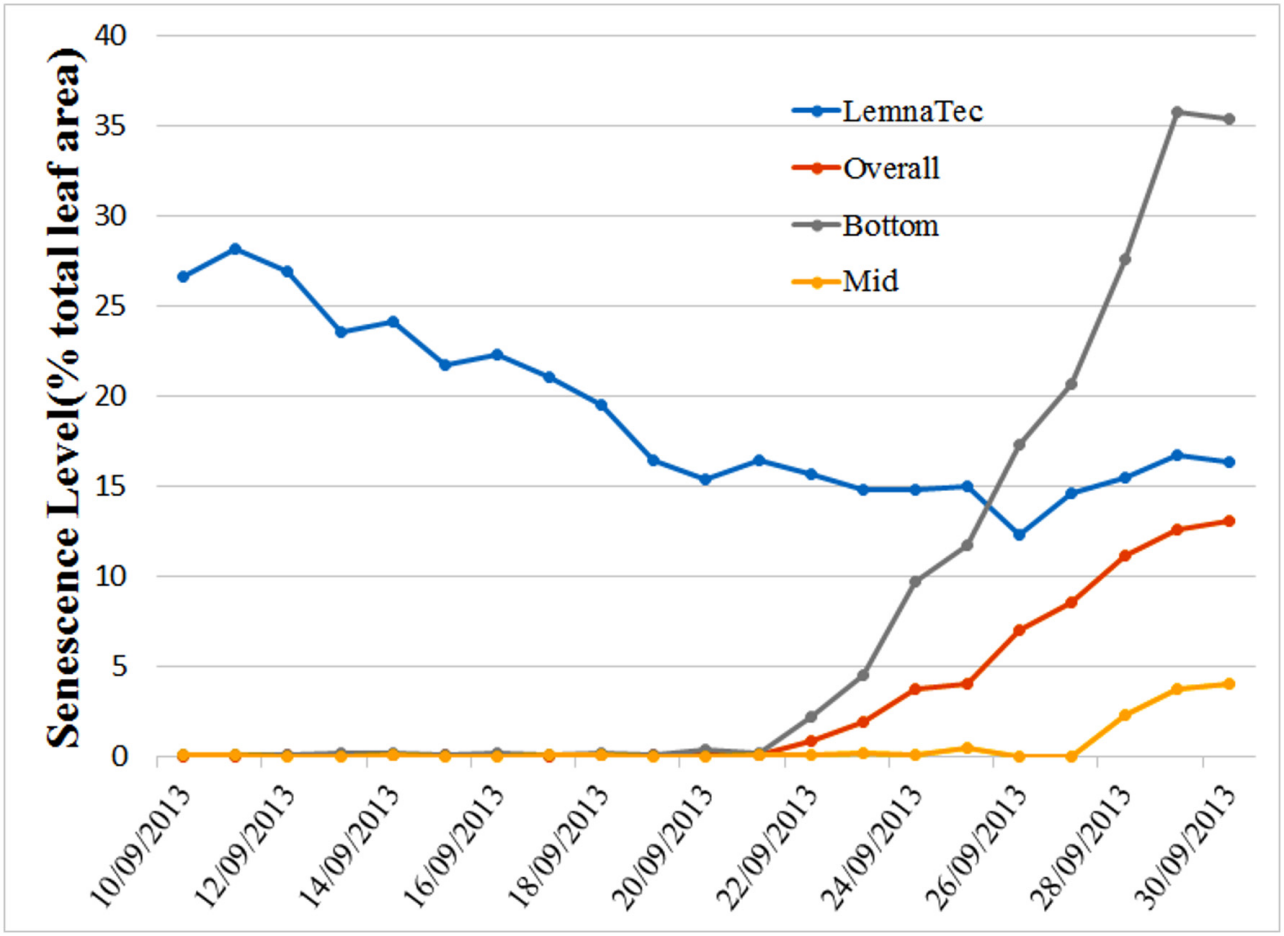
Comparison of senescence estimations using the method proposed here (curves denoted Overall, Mid and Bottom) and a direct application of the color analysis software provided by The Plant Accelerator’s LemnaTec imaging system. For the Lemnatec results, only a whole-of-plant measure is available with which we compare a corresponding measure, which in turn is broken down into the senescence levels determined in the bottom and middle zones.

It should come as no surprise that all images exhibit some degree of blurring and color distortion. These effects can be substantial if the camera setting is far from optimal and if the plants being imaged are small; color distortion effects, extending across several pixels orthogonal as well as along leaf boundaries, can be significant. With the view to applying our method in high throughput facilities. It makes sense to compare the outcome of our analysis with the options currently available in such systems. Accordingly, we consider results using a typical system’s in-built, color analysis software (in the present case, The Plant Accelerator’s LemnaTec imaging system software). A direct application on the original images gives an estimated senescence level, measured as a percentage of whole plant area, of greater than 10%, even for initial images in the sequence known not to exhibit any senescence. A quantitative comparison of results using our method with those of a direct color scheme analysis applied to a specific plant image series is shown in Fig 7. In the case of our method, the results include an “Overall” measure (whole plant) and two separated measures, “Mid” and “Bottom”, to indicate that onset appears primarily (though not always) in the bottom zone. Results of the LemnaTec system is not separated so only the “Overall” measure is given.

It is clear from Fig 7 that a direct application of color analysis results in a significant error. Two facts emerge from that analysis: first, when the plant is young and therefore small, blurring and color distortion significantly distorts the senescence estimation; second, when actual senescence is present and significant, the estimated level may qualitatively mimic the true development, but (a) is quantitatively overestimated and (b) cannot be used to establish the point of onset.

**Fig 8:**
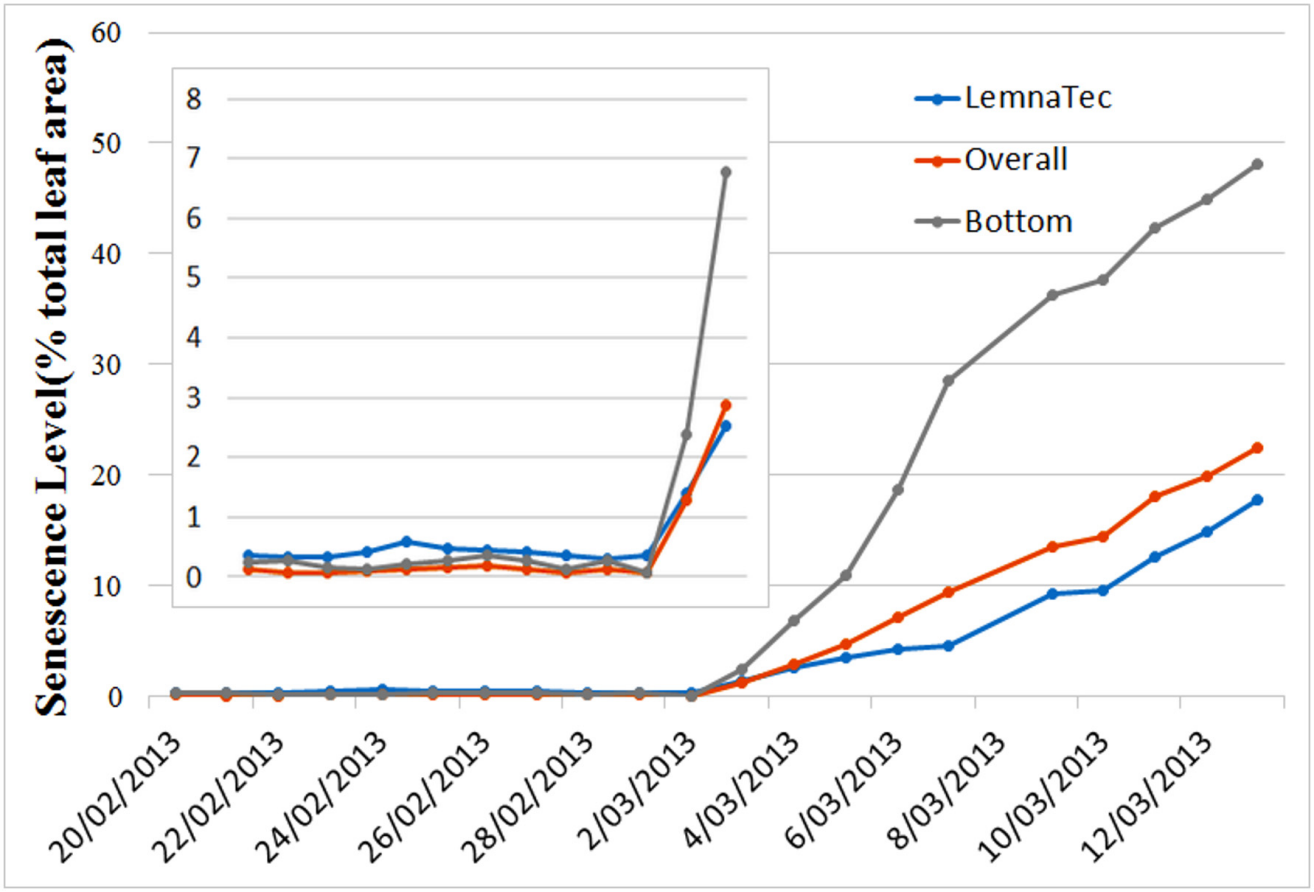
Comparison of senescence estimations using the method proposed here (curves denoted Overall and Bottom) and the application of a machine-learned, color analysis provided by The Plant Accelerator’s LemnaTec imaging system (curve labelled “LemnaTec”.

A recent LemnaTec software upgrade offers the user the option of using machine learning methods to learn color differences between typical plant greens, recurring background colors and colors associated with senescent leaves as well as the colors (usually light yellow) caused by burring and color distortion. This refinement dramatically improves the accuracy of the in-built tool. An updated comparison for the same image sequence is given in Fig 8; the difference between the two analyses is significantly reduced (the bias is now between 2.0% and 5.0%). To be more precise, when the plants are young, possessing small green leaves, the machine learning result slightly overestimates the senescence level (see figure inset), which is enough to eliminate any possibility of using this method to detect the onset of senescence. The disparity between the estimated senescence levels becomes greater when there is a significant level of senescence present. The disparity is due to the machine learning procedure itself: the color subspaces associated with the yellow due to blurring and color distortion and the yellow of plant senescence overlap. It is therefore inevitable that some parts of senescent leaves will be classified as background, resulting in an underestimation of the level of senescence actually present.

It is important also to point out that although there is a clear improvement in the LemnaTec system’s senescence estimation, it comes at a price and with limitations. The price is that some manual labelling of images is required for training of the machine learning algorithm. This detracts from the use of this solution for high throughput applications. As with all machine learning techniques, another limitation is that the learning step, which is valid for one experiment, may not be valid for another experiment, even for the same plant species, if the camera settings differ.

**Fig 9:**
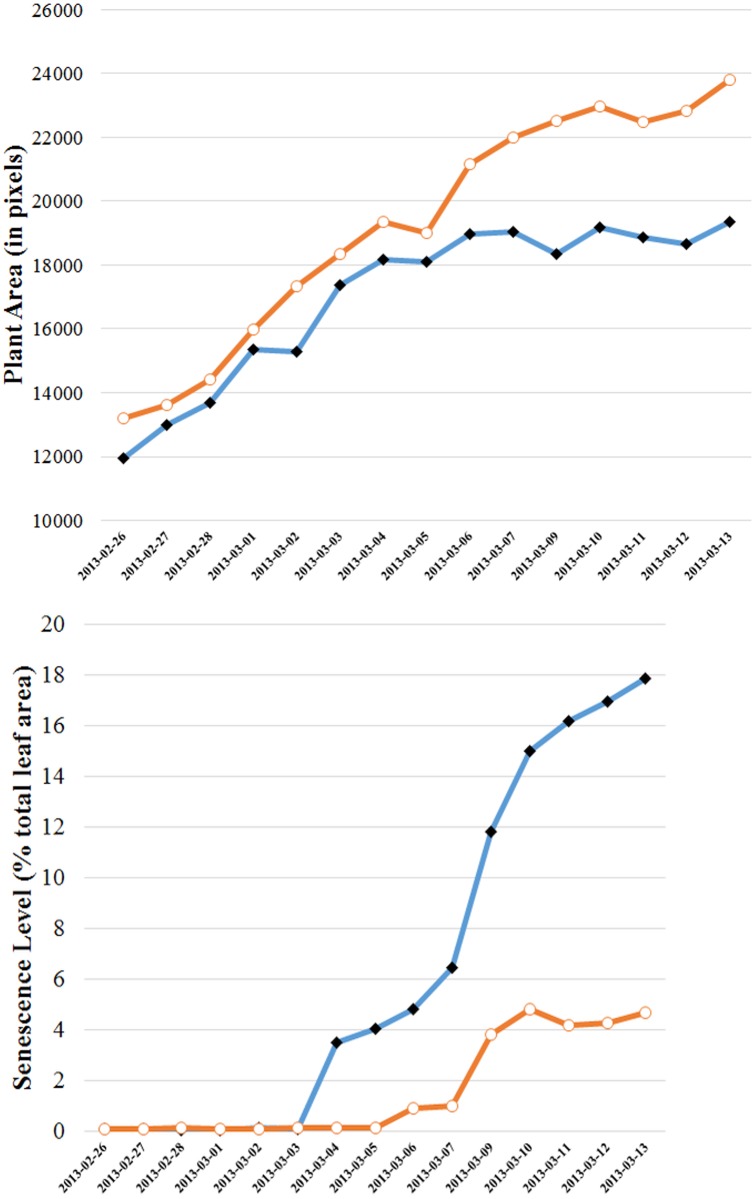
Whole-of-plant assessment of growth (plant area) and senescence as a function of time for two individual plants under N2, chosen arbitrarily. Top figure features the time developments of total projected plant area (all leaves and stems) for the two plants, depicting similar growth behavior. Bottom figure shows the percentage of senescence present in the leaves of these plants relative to their total plant area. The two individual plants exhibit different rates of senescence development as well as different onset dates.

In Fig 9 we summarize the overall measures for two individual plants over the 15 days that images were taken. Two time series are shown. Fig 9(a) features the time series of total visible plant area for the two plants, while Fig 9(b) highlights the percentage of senescence (yellow and brown color categories) present relative to total plant area as a function of time.

The regular imaging of plants (particularly imaging from several perspectives at once) over a significant period of time offers the potential for the time-course capture of a significant amount of information on a number of important phenotypic traits. Realizing that potential cannot be achieved using either subjective means or inadequate processing tools. Fig 9 highlights the possibility of realizing the potential with the application of our color correction and classification procedure to quantify traits such as plant growth over time. What is particularly clear from Fig 9(b) is that the development of plant senescence and its dependence on applied stress can now be quantified rigorously. Indeed, two specific features of the senescence process can be quantified, namely, the day on which senescence first appears (onset) on an individual plant (and where) and the rate at which senescence progresses, either absolutely or as a relative percentage of leaf area. Moreover, the latter information can be refined into zones for a detailed study of senescence.

**Fig 10:**
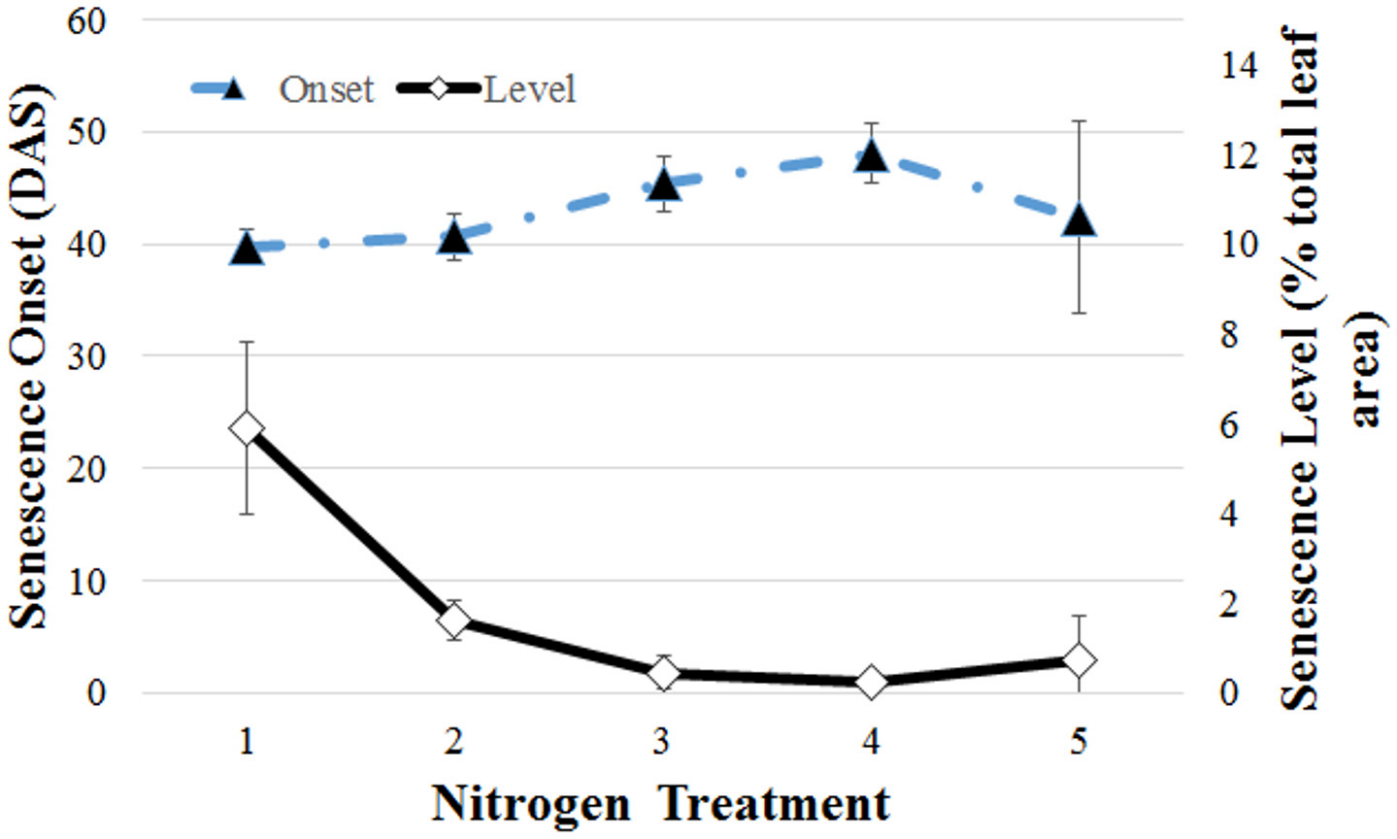
Summary of senescence dependence on nitrogen treatment (*N*1 − *N*5, horizontal axis) for the Gladius wheat plant variety. Shown are mean values of onset determination (days after sowing, DAS, dashed curve and solid triangles, left vertical axis) and final degree of senescence (percentage of total project leaf area, solid curve and open diamonds, right vertical axis). Error bars show the variation across three repeats.

These two specific features are exemplified in Fig 10, which summarizes the effects of nitrogen treatment on both the time of onset and on the final degree of senescence, the latter relative to the total projected leaf area. Only one wheat variety (the genotype Gladius) is represented, with results averaged over a number of repeats. The error bars therefore refer to variation over the repeats and are not indicative of errors in senescence estimation. A more extensive study comparing plant responses to nitrogen across a range of genotypes is the subject of a separate publication. Although the Gladius variety appears less sensitive to nitrogen level than do other varieties, it is nevertheless evident that the method is able to detect even minor variations with added nitrogen for this genotype. The delay of onset seen here with application of medium levels of nitrogen agree with previous observations. The results in Fig 10 also demonstrate that our method can quantify the final proportion of senescent leaves relative to the total leaf area, reflecting the stasis in senescence relative to total leaf mass that manifests with the addition of nitrogen.

#### Chickpea pilot experiment

The experiment on chickpea plants represents a pilot study of the effects of salt stress as well the influence of soil condition. This experiment exemplifies a common case where only post-processing of an image sequence is possible, and where the resolution of plant images is satisfactory to quantify some features but is not sufficiently high to assess plant senescence. In these images, the color distortion between two pixels along leaf boundaries is significant. Application of the in-built color analysis software on the original images estimated a senescence level, measured in terms of percentage of senescence to whole plant area, of greater than 10% even for the first images in the sequence, of young plants exhibiting no senescence. An application of our color correction procedure followed by a color analysis on the recovered images found an estimated senescence level of less than 1.0%, while the in-built, machine-learned color analysis tool estimated a senescence level of around 2.0% for these first images.

To further evaluate the performance of our color correction procedure, our results were compared with those of manual inspection based on the 1–10 scale [18] described in the Materials and Methods section. Note that the manual inspection was undertaken 2 and 3 days after the last imaging day. The results of both the subjective and the objective means of quantifying senescence level are presented for comparison in Table 1.

**Table 1:** Evaluation of the performance of our senescence analysis algorithm as applied to chickpea images.

Treatment	Our method		Manual (after 2 days)		Manual (after 3 days)	
	(Percentage of senescent leaves)		(Senescence score)		(Senescence score)	
(mM NaCl)	Genesis 836	Rupali	Genesis 836	Rupali	Genesis 836	Rupali
0	0.73	1.64	1	4	3	4
	0.53	8.03	1	5	2	6
	3.30	1.08	2	2	4	5
	0.53	2.27	1	4	3	3
30	1.14	3.69	2	5	5	5
	0.34	5.65	1	4	4	5
	0.91	11.81	2	7	3	10
	0.84	1.71	2	4	3	7
40	0.71	3.13	2	4	5	5
	1.05	9.80	2	7	5	8
	0.91	11.81	2	4	3	6
	0.78	10.15	2	8	3	10
60	0.99	1.40	4	5	5	5
	0.50	2.77	1	5	4	5
	0.81	2.70	1	5	4	10
	1.81	25.75	2	8	3	10

In this table our estimated level or percentage of leaf senescence is based on the last image in the sequence of 8 chickpea plant images.

**Fig 11:**
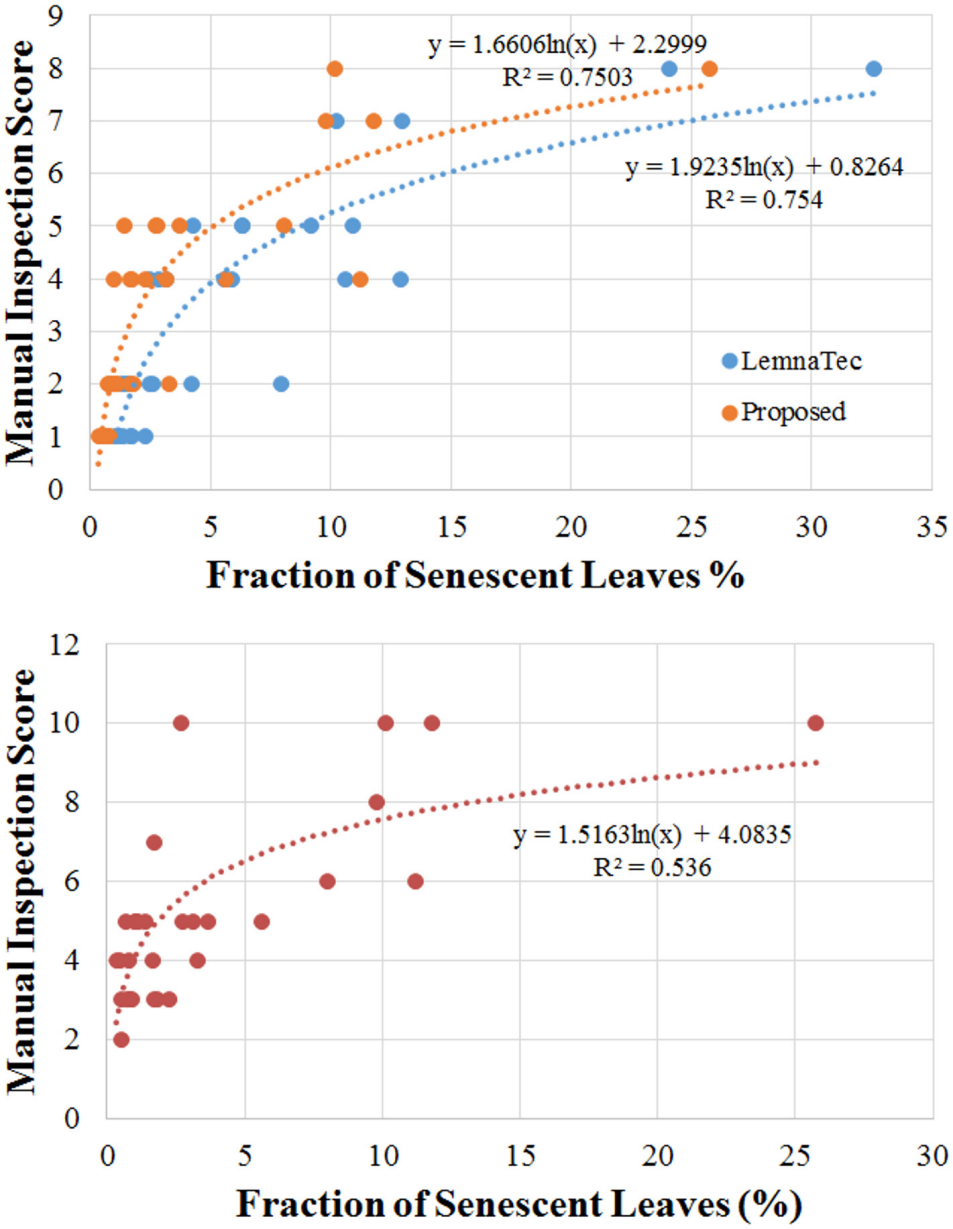
Scatter plots of manual inspection scores versus the objectively estimated senescence measure proposed in this paper and that of the machine learning method adapted by the LemnaTec system for a set of chickpea plants. Manual scoring was performed on two occasions: 2 days after the last imaging day (top panel) and 3 days after the last imaging day (bottom panel).

Despite the two diametrically contrasting measures of senescence, there remains a definite correlation between the outcomes, even though the manual inspections were conducted 2 and 3 days after the final images were taken. This is demonstrated quite convincingly in Fig 11, which exhibits a highly correlated functional (logarithmic) relationship between the two measures; the R-squared values are 0.7503 (0.754 in the case of the LemnaTec estimate) and 0.536, respectively, for the manual inspection 2 and 3 days after the last image was taken. A decreasing R-squared value is expected with increasing time difference between the day of imaging and the day of manual inspection. What is not yet clear from the results obtained so far (Fig 11), is whether a more refined manual inspection score and more frequent inspections, to compare with a simultaneous objective senescence measure, will add meaning to the fitted functional relationship. Unfortunately, manual inspection is time consuming, costly and highly subjective, which only highlights the effectiveness of the automated and objective process proposed here.

**Fig 12:**
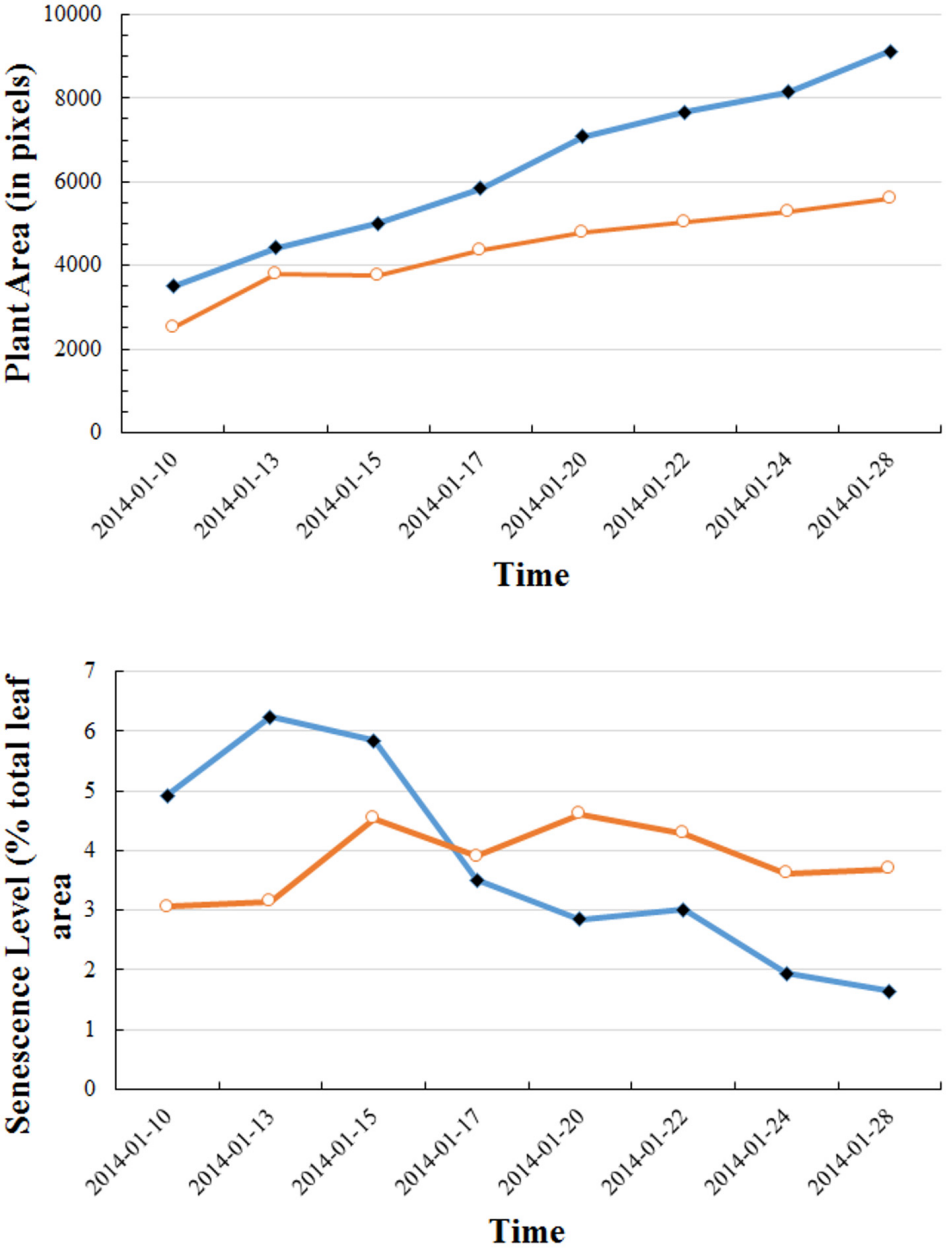
Whole-of-plant assessment of growth (plant area) and senescence as a function of time for two individual chickpea plants. Top figure shows the time developments of total projected plant area (all leaves and stems) for the two plants, depicting qualitatively similar but quantitatively different growth behavior. Bottom figure shows the percentage of senescence present in the leaves of these plants relative to their total plant area. The two individual plants exhibit different rates of senescence development.

**Fig 13:**
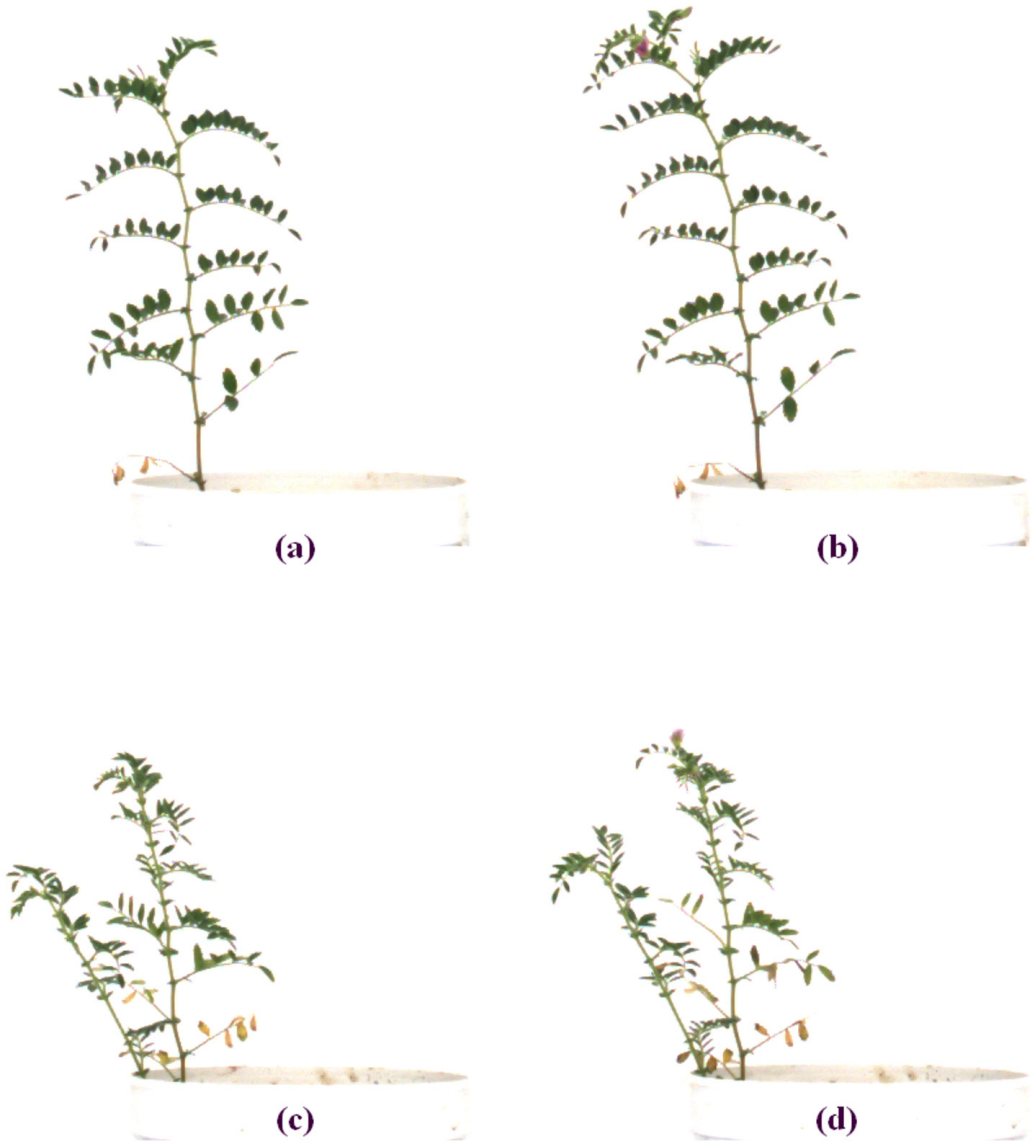
Two examples of senescence patterns. (a) and (b) depict similar sized leaf areas of the same plant on different days; in (b) the plant has grown a little larger. Figs (c) and (d) show the same plant (different from (a) and (b)) on different days but at a much later stage of development when more leaves have become senescent and after some senescent leaves have fallen off.

As with the wheat experiment, by utilizing the regularly taken sequence of chickpea plant images we are able to track both the growth behavior as well as the senescence process over time. In Fig 12 we demonstrate this functionality based on the time series of images for two individual plants. Although the growth behavior, in terms of projected plant area (all types), is similar to wheat in the sense of increasing with time, in contrast to the wheat experiment, the analysis reveals a decreasing trend in senescence of one plant and a semi-steady, but fluctuating behavior for the other plant. The differences can be attributed to the genotypic responses of the two plants under salt stress: for one plant (blue curve), the senescence pattern did not spread over the plant during the plant’s continual growth, leading to a decreasing fraction of leaf senescence. This can be verified by appeal to the image comparison in Fig 13(a) and 13(b). In contrast, for the other plant (red curve) senescence increased with time, but leaf fall resulted in a significant fluctuation in the percentage of leaves exhibiting senescence. This is exemplified in Fig 13(c) and 13(d).

## Conclusions and Future Work

In plant phenomics it is not only those characteristics associated with the plant growth phase that are important factors to capture to assess a plant’s performance against growth conditions, *i.e.*, a plant’s stress response. It is equally important to quantify traits indicative of a plant’s age and ill health, such as senescence. In this paper we have proposed a fully automated algorithmic tool for senescence estimation, encompassing the restoration of color, plant foreground segmentation and a color classification for senescence analysis. One less obvious outcome of our efforts is the demonstration that manual inspection is inadequate as a means of assessing the senescence state of a plant. Image analysis based on color differentiation is a sound alternative. However, under high throughput conditions where the entire plant is imaged, the resolution may be insufficient to accurately capture senescence onset and development. Another related major problem is the absence of adequate camera color calibration. The approach we advocated here addresses, in a practical way, these major problems. As a result, we not only can reduce image color distortion but also improve image quality sufficiently to quantify senescence accurately. To verify the procedure’s effectiveness, we compared our results with senescence scores attained by manual inspection and the senescence levels estimated by the in-built tool. We found that there is a correlation between the these measures, which no doubt has its origins in the fact that these measures are based on plant appearance.

It is worth reiterating that the process of labelling or annotating of images for the training of a machine learning approach is time consuming, with the final result intrinsically dependent on the quality of annotation. As mentioned already, machine learning approaches have their limitations, which can prevent them from being used in high throughput applications. In contrast, the philosophy of the approach proposed here is inherently high throughput, and whose full automation can be reinforced by using, as benchmark, the image of a standard color chart at the beginning of an experiment, instead of estimating an undistorted image of a young plant.

One direction for further study is to undertake a more extensive comparison between the manual and the automated approaches to identify (if possible) a reason for the observed functional form for this correlation. A second direction for further study was identified in the chickpea study, where both the decreasing senescence trend and the fluctuating behavior of the two curves in Fig 12(b) indicate that a detailed and aggregated monitoring of leaf senescence, including the (time) tracking of leaf fall, is required for a complete assessment of senescence for this phenotypic trait to properly characterize a plant’s stress response.

## Supporting Information

S1 Appendix Detailed description of the segmentation process. (PDF)
