# Supplementary material for: Quantifying the Onset and Progression of Plant Senescence by Color Image Analysis for High Throughput Applications
Source: PLoS One. 2016 Jun 27;11(6):e0157102. doi: 10.1371/journal.pone.0157102 (PMC4922665; doi:10.1371/journal.pone.0157102)
Supplement: S1 Appendix — (PDF) [file pone.0157102.s001.pdf]

PLOS

Quantifying the onset and progression of plant senescence by color image analysis for high throughput applications

Jinhai Cai, Mamoru Okamoto, Judith Atieno, Tim Sutton, Yongle Li, Stanley J. Miklavcic

S1 Appendix

Automated Plant Segmentation

The segmentation between plants and the background is a difficult task for many reasons, particularly the background is consisting of several objects in the greenhouse such as the pot and the supporting frame. The change of plant colors is the only constant thing during the whole life cycle of plants. It is expected that the color of senescent leaves will be similar to the color of pot rim, where the dirt on the pot rim is yellow as illustrated in S1 Fig(a). Clearly the color space of pot rims and that of plants are overlapped, thus significant segmentation error is inevitable if the segmentation algorithm is based on color analysis. However, this problem can be solved if we known the background.

The estimation of background

There are many algorithms developed for the estimation of background [1]. Most of them assume that the background is still and the foreground is moving. However, in this case, plants cannot move to other locations instead they are growing. These background estimation algorithms are not suitable for our experiments. The LemnaTec commercial software uses a pot with no plant in for background estimation. This approach is useful in reducing the segmentation error. However, pots are different from each other due to different labels and rim colors.

In all our experiments conducted in the greenhouse, plants are very young and green on the first imaging day. This allow us to estimate the background by removing green areas and replacing with surrounding background in the images from the first imaging days. In some experiments, a blue frame is used to support the plant in a pot from falling. The position of a blue frame in a pot can be changed by the plant during its life cycle, so it is not treated as a part of the background. We use the same way to remove the frame by replacing blue areas with surrounding background. S1 Fig(b) shows an example of the background estimation.

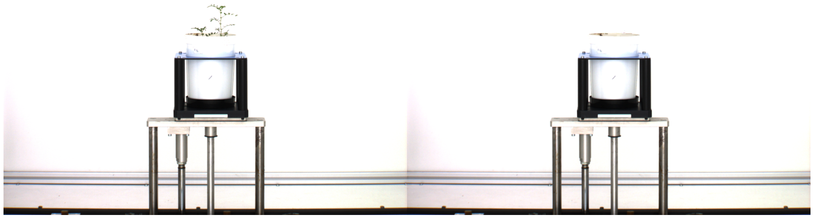

S1 Fig. The estimation of the background. (a) is the original image; and (b) is the estimated background.

The segmentation

With the estimated background, it is common to segment the foreground by subtracting the background and then thresholding. In the greenhouse, the convey system sends the pot to almost the same location for imaging. However, there is a small disparity, which result in a small shift of the pot in the image. In order to handle the small shift, we calculate the modified difference between the image and the estimated background and then apply the thresholding. The modified difference is defined as

$$d_m(x,y) = min_{i=-t,j=-t}^{i=t,j=t}|I(x,y) - B(x+i,y+j)|, \quad \text{(S1)}$$

where $I(x, y)$ is the pixel of the image at (x, y), $B(x, y)$ is the pixel of the estimated background at (x, y) and t is a value for the small shift in images. S2 Fig shows an example of the segmentation results. The segmentation results are pretty good but not perfect as very small areas of the blue frame are segmented as plants.

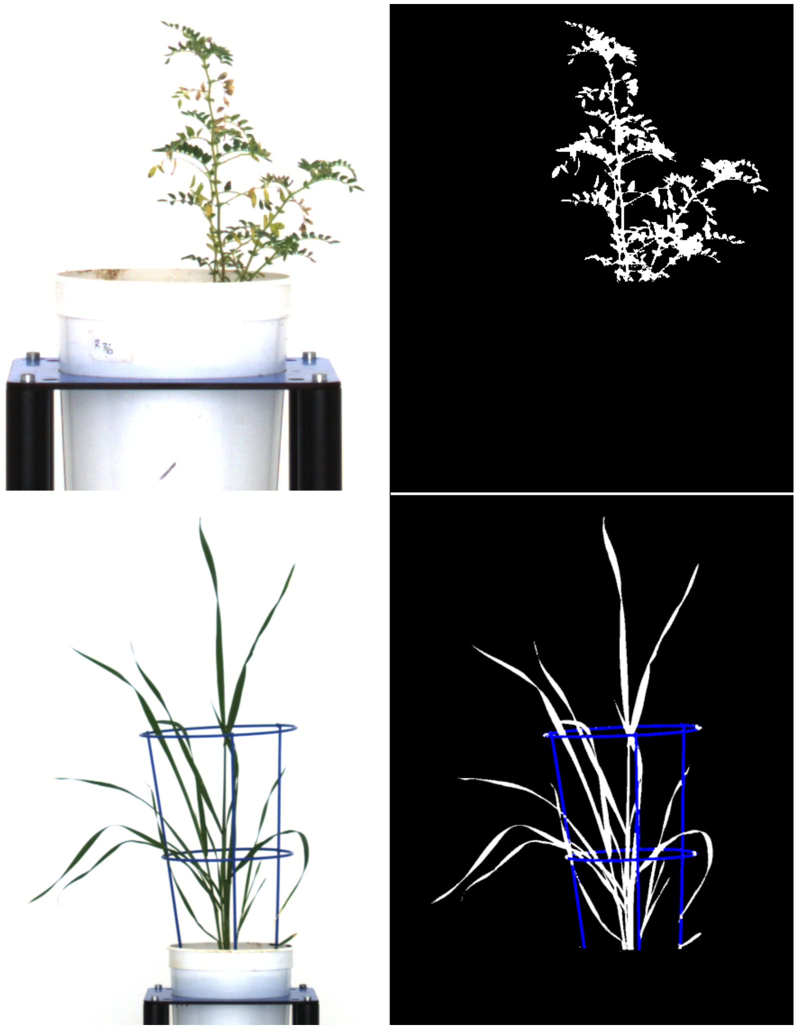

S2 Fig. The image segmentation. Left images are the original images and right images are the segmented images, where the parts in blue color are the blue frames.

Color Classification

The color classification is relatively straightforward. We selected two segmented images of plants, one at a relatively early growth stage so that most leaves are with dark green and light green color and another plant with significant senescence so that some leaves

are with light yellow color and some leaves (dead leaves) are with brown color. We labelled these two images with four color categories. The color classification can be conducted by the k-Nearest Neighbours (kNN) algorithm [2]. In order to reduce the computational cost, we apply the k-means clustering algorithm [3] to form clusters and we use the cluster centres instead of directly using pixels in the labelled images as color feature points in the kNN algorithm. In our experiments, the number of color clusters for plants is set to 18 as there is no visible difference from using the two labelled images directly. At the same time, the computational cost is significantly reduced.

References

1. Sobral A, Vacavant A. A comprehensive review of background subtraction algorithms evaluated with synthetic and real videos. Computer Vision and Image Understanding. 2014;122:4 – 21.
2. Altman NS. An introduction to kernel and nearest-neighbor nonparametric regression. The American Statistician. 1992;46(3):175–185.
3. Kanungo T, Mount DM, Netanyahu NS, Piatko CD, Silverman R, Wu AY. An efficient k-means clustering algorithm: Analysis and implementation. IEEE Transactions on Pattern Analysis and Machine Intelligence. 2002;24:881–892.
